# Dietary Acid Load Across Vegans, Lacto-Ovo-Vegetarians, Fish-Eaters, and Meat-Eaters, and Its Association with Body Mass Index: A Secondary Analysis from an Italian Survey (The INVITA Study)

**DOI:** 10.3390/foods15152613

**Published:** 2026-07-26

**Authors:** Luciana Baroni, Davide Catania, Chiara Bonetto, Alexey V. Galchenko, Giada Guidi, Nicola de Bortoli

**Affiliations:** 1 Scientific Society for Vegetarian Nutrition—SSNV, 30171 Venice, Italy; luciana.baroni@scienzavegetariana.it (L.B.); alexey.galchenko@scienzavegetariana.it (A.V.G.); giada.guidi@scienzavegetariana.it (G.G.); 2 Department of Theoretical and Applied Sciences—DISTA, University eCampus, 22060 Novedrate, Italy; 3 Section of Psychiatry, Department of Neuroscience, Biomedicine and Movement Sciences, University of Verona, 37134 Verona, Italy; chiara.bonetto@univr.it; 4 Earth Philosophical Society “Melodia Vitae”, San Clemente, CA 92673, USA; 5 Division of Gastroenterology, Department of Translational Research and New Technologies in Medicine and Surgery, University of Pisa, 56126 Pisa, Italy; nicola.debortoli@unipi.it; 6 NUTRAFOOD, Interdepartmental Center for Nutraceutical Research and Nutrition for Health, University of Pisa, 56126 Pisa, Italy

**Keywords:** plant-based diets, PBD, vegetarian, lacto-ovo-vegetarian, LOV, vegan, dietary acid load, PRAL, NEAP, BMI

## Abstract

Diet represents a key determinant of the body’s acid–base balance because foods are metabolized into non-volatile acids or bases. An increase in dietary acid load can elicit low-grade metabolic acidosis, which has been associated with systemic inflammation and the risk of adverse cardiometabolic conditions, including obesity. Potential renal acid load (PRAL) and Net Endogenous Acid Production (NEAP) represent the major markers of dietary acid load. The aim of this secondary analysis was to compare the acid load of four different diets and investigate its association with Body Mass Index (BMI) in a sample of the general population. In an online survey, the INVITA study, four dietary patterns (meat-eaters, fish-eaters, lacto-ovo-vegetarians, and vegans) were compared for their corresponding PRAL and NEAP values, and the associations of both parameters with BMI were evaluated. Multiple linear regression models were progressively adjusted for dietary pattern, energy intake, age, sex, physical activity, smoking status, and education level. The stepwise decrease in animal food consumption across the four dietary patterns was associated with a progressive reduction in dietary acid load, with vegan participants showing the lowest values. While PRAL did not prove consistently associated with BMI in this cohort, NEAP remained significantly associated with BMI even after full adjustment for lifestyle confounders (β = 0.032, *p* = 0.00013). However, because NEAP includes an anthropometric component based on body surface area, this association should be interpreted cautiously, as it may partly reflect mathematical coupling rather than a purely diet-derived effect. PRAL should be considered the more diet-specific marker, while NEAP represents a composite index combining dietary acid load and anthropometric scaling. Because PRAL and NEAP do not account for protein origin, future studies should assess whether diet-specific reference ranges could improve their comparisons for dietary patterns including mostly or exclusively plant protein.

## 1. Introduction

Diet is a primary determinant of the body’s acid–base balance, as various nutrients are metabolized into non-volatile acids or bases [1,2]. Protein serves as one of the major nutrients driving the dietary acid load, particularly due to the sulfur-containing amino acids methionine and cysteine. In the human body, the sulfur moiety within these amino acids is ultimately oxidized to sulfate, yielding sulfuric acid—the strongest endogenous acid produced via metabolism [3]. Excess phosphate, representing phosphoric acid, constitutes another significant source of systemic acid load. Although phosphate is essential for the formation of the renal phosphate buffer system that excretes excess protons into the urine, phosphorus deficiency states are currently rare in developed countries. Conversely, overnutrition and the widespread incorporation of phosphate-based food additives deliver substantial amounts of phosphorus to the human body [4]. In contrast, the primary nutrients that mitigate the dietary acid load are alkaline and alkaline earth metals, specifically potassium, calcium, and magnesium. These minerals are predominantly ingested as cations of organic salts, which are subsequently metabolized into bicarbonate, thereby replenishing the carbonate buffer system. Vegetarians (particularly vegans [VNs]) consume significantly higher amounts of potassium and magnesium. Regarding calcium intake, lacto-ovo-vegetarians (LOVs) frequently emerge as leaders due to their high consumption of dairy products, although suboptimal calcium intake remains prevalent across all dietary groups [4]. On the other hand, meat-eaters (MEs) consume greater amounts of both total protein and sulfur-containing amino acids, as we found in our previous study [5]. Notably, significant differences in total phosphorus intake between dietary groups are rarely observed [4], and our recent studies yielded similar results [6,7]. As previously stated, phosphorus ingestion depends heavily on the utilization of phosphate additives by specific food manufacturers, rendering its precise quantification in dietary assessments exceptionally challenging [4].

Growing attention has been paid to the role of dietary acid load in human health, because its increase can elicit low-grade metabolic acidosis, which has been associated with systemic inflammation and the risk of adverse cardiometabolic conditions, including obesity [8,9,10,11,12].

Potential renal acid load (PRAL) and Net Endogenous Acid Production (NEAP) represent the major markers of dietary acid load. PRAL indicates a food’s acid- or base-forming potential and is based on the values of the five nutrients that mainly influence pH (phosphorus, proteins, potassium, calcium, and magnesium). Sodium, along with chloride, is absent from the PRAL calculation formula, as these ions are ingested almost exclusively in the form of table salt (NaCl), where they are represented equimolarly and, therefore, mathematically cancel each other out in the equation. Furthermore, the dietary intake of sodium and chloride is primarily driven by individual salt-adding habits and taste preferences rather than the specific type of dietary pattern [4]. NEAP, further adjusting PRAL for body surface area (calculated on weight and height), allows an appropriate prediction of the effects of diet on urine acidity [13].

The aim of this secondary analysis was to compare the acid load of four different diets (vegan [VN], lacto-ovo-vegetarian [LOV], fish-eater [FE], and meat-eater [ME]) and investigate its potential association with Body Mass Index (BMI, kg/m^2^) in a sample of the general population.

## 2. Materials and Methods

### 2.1. Data Collection

The INVITA study (INVestigation on ITAlians’ habits and health) is an online survey launched on 26 July 2022 to gather cross-sectional information on the lifestyle, health status, and diet of the Italian general population. Data presented in this article represent a secondary analysis of data not analyzed in our previous article, describing the nutrient intakes of the sample [6]. Online questionnaires were administered by the Scientific Society for Vegetarian Nutrition (SSNV-APS), an Italian non-profit organization, through a dedicated web application hosted at www.studioinvita.it (accessed on 26 July 2022) and accessible via computers, tablets, and smartphones. Participants took part voluntarily and anonymously in the survey by a link promoted through social media and newsletters. Informed consent was obtained from all participants. All data were self-reported. Non-inclusion criteria were: age under 18 years, pregnancy or breastfeeding, and adherence to restrictive plant-based dietary patterns (e.g., macrobiotic, fruitarian, raw-food, or hygienist diets). Precisely, participants who self-reported following a restrictive diet were identified based on responses to five closed-ended screening questions, resulting in the exclusion of 11 subjects in total.

Non-inclusion criteria were applied by the software prior to the download. The collected data were downloaded and managed by designated data management personnel who could not identify individual study participants.

Diet composition data were collected by a 3-day weighed online food questionnaire, including one weekend day. Foods were selected from a predefined list derived from the BDA database of the Italian European Institute of Oncology [14] and the USDA nutrient database [15], and participants self-reported the amount consumed for each item—including added salt, sugar, and oil—as well as the meal in which it was eaten (for further details on the databases, please consult Baroni, 2024 [6] (see Supplementary Tables S1 and S2 of that study).

Sociodemographic characteristics and lifestyle habits were also collected using ad hoc forms. The variables assessed included gender, age, marital status, educational level, occupation, self-reported height and weight, smoking status, monthly alcohol consumption, physical activity, prevalence of menopause (only for females), and the age at menopause. Body Mass Index (BMI) was calculated as weight in kilograms divided by height in meters squared (kg/m^2^). Alcohol consumption was expressed in alcohol units (AUs), with one AU corresponding to 12 g of pure alcohol, approximately equivalent to 330 mL of beer, 125 mL of wine, 80 mL of vermouth, or 40 mL of spirits. At-risk alcohol consumption was defined as more than 60 AUs per month for males and more than 30 AUs per month for females [16,17]. Smoking status was recorded as a dichotomous variable (yes/no). Physical activity was expressed in levels of physical activity (PAL) according to the Report of the Joint FAO/WHO/UNU Expert Consultation, which categorizes PAL in 5 classes: (a) very low activity (<1.40); (b) sedentary or light activity lifestyle (1.40–1.69); (c) active or moderately active lifestyle (1.70–1.99); (d) vigorous or vigorously active lifestyle (2.00–2.40); and (e) very high activity (>2.40) [18].

The study protocol was reviewed and approved by the Bioethics Committee of the University of Pisa, Italy (Prot. No. 0116339/2021; approval date: 29 September 2021).

### 2.2. Data Assessment

**Figure 1:**
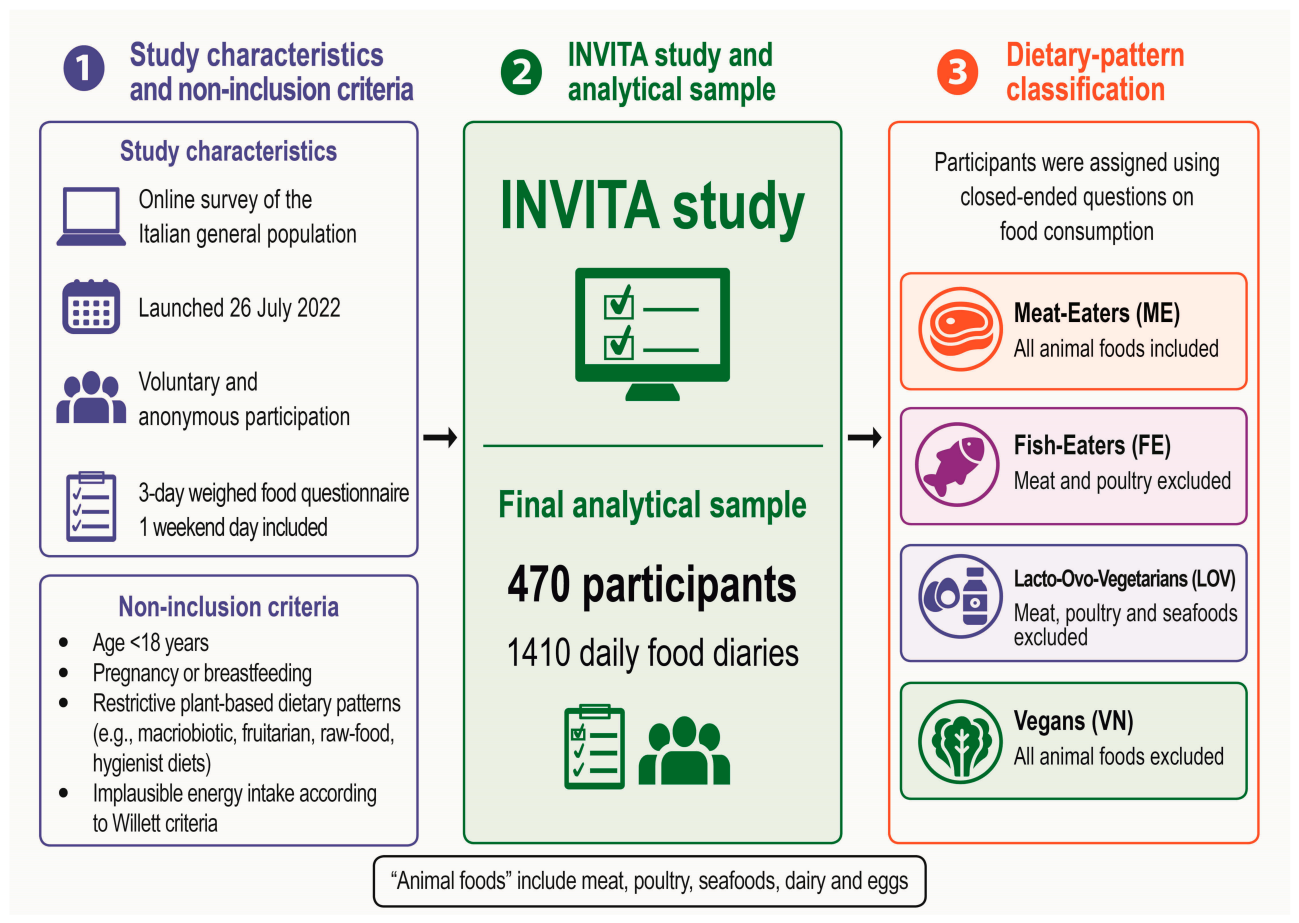
The INVITA study: study characteristics, non-inclusion criteria, analytical sample and dietary pattern classification.

Dietary patterns were classified, based on four closed-ended questions on food consumption (Y/N), and participants were assigned to one of four dietary groups: meat-eater (ME), fish-eater (FE), lacto-ovo-vegetarian (LOV), and vegan (VN) (Figure 1).

In this secondary analysis, the nutrient and anthropometric data of interest were used to calculate the dietary acid load as follows [19,20]:

- PRAL mEq/d = (0.49 × protein g/d) + (0.037 × phosphorus mg/d) − [(0.021 × potassium mg/d) + (0.026 × magnesium mg/d) + (0.013 × calcium mg/d)].
- NEAP mEq/d = PRAL + OA_est_^a^
  ^a^OA_est_ (mEq/d) = Body surface area^b^ × 41/1.73
  ^b^Body surface area (m^2^) was calculated according to the formula of Du Bois and Du Bois: 0.007184 × height (cm)^0.725^ × weight (kg)^0.425^ [21].

### 2.3. Statistical Analysis

The four dietary patterns were compared for the corresponding PRAL and NEAP values.

Preliminary assessment of the normality of distributions was conducted by analyzing skewness within dietary groups. Because PRAL and NEAP showed non-normal distributions, non-parametric statistical tests were applied. Continuous variables are presented as medians (Me) and interquartile ranges (IQRs). Differences among dietary groups were assessed using the Kruskal–Wallis test, followed by Dunn post hoc pairwise comparisons with Holm correction.

Moreover, associations between dietary acid load indices and BMI were evaluated using Spearman correlation coefficients with Holm correction for multiple testing.

Multiple linear regression analyses were performed to evaluate the independent association between dietary acid load (PRAL and NEAP, entered as continuous variables) and BMI. Because PRAL and NEAP are highly correlated indices of acid–base balance, they were evaluated in separate models to avoid multicollinearity. A progressive adjustment strategy was adopted to assess the robustness of the associations. Basic models (Model 1) were adjusted for total energy intake and dietary pattern. Fully adjusted models (Model 2) additionally included age, sex, physical activity level (PAL), smoking status, and education level, to account for potential lifestyle and demographic confounders. Complete case analysis was performed for participants with available data for all covariates. Results were expressed as β coefficients with 95% confidence intervals (CI).

All statistical tests were conducted using two-sided hypotheses, with statistical significance set at *p* < 0.05. Statistical analyses were performed in Python 3.12.13 (Python Software Foundation, Wilmington, DE, USA) using Google Colab (Google LLC, Mountain View, CA, USA), with the Pandas 2.2.2 (NumFOCUS, Austin, TX, USA), Scipy 1.16.3 (NumFOCUS, Austin, TX, USA), Statsmodels 0.14.6 (NumFOCUS, Austin, TX, USA), and Scikit-Posthocs 0.13.0 (Open Source) Libraries.

A post hoc power analysis was conducted using G*Power software (version 3.1; Heinrich Heine University Düsseldorf, Düsseldorf, Germany; *F* tests, linear multiple regression: fixed model, *R*^2^ deviation from zero; significance level α = 0.05; total sample size *n* = 470) to evaluate the statistical robustness of the four estimated models.

## 3. Results

**Figure 2:**
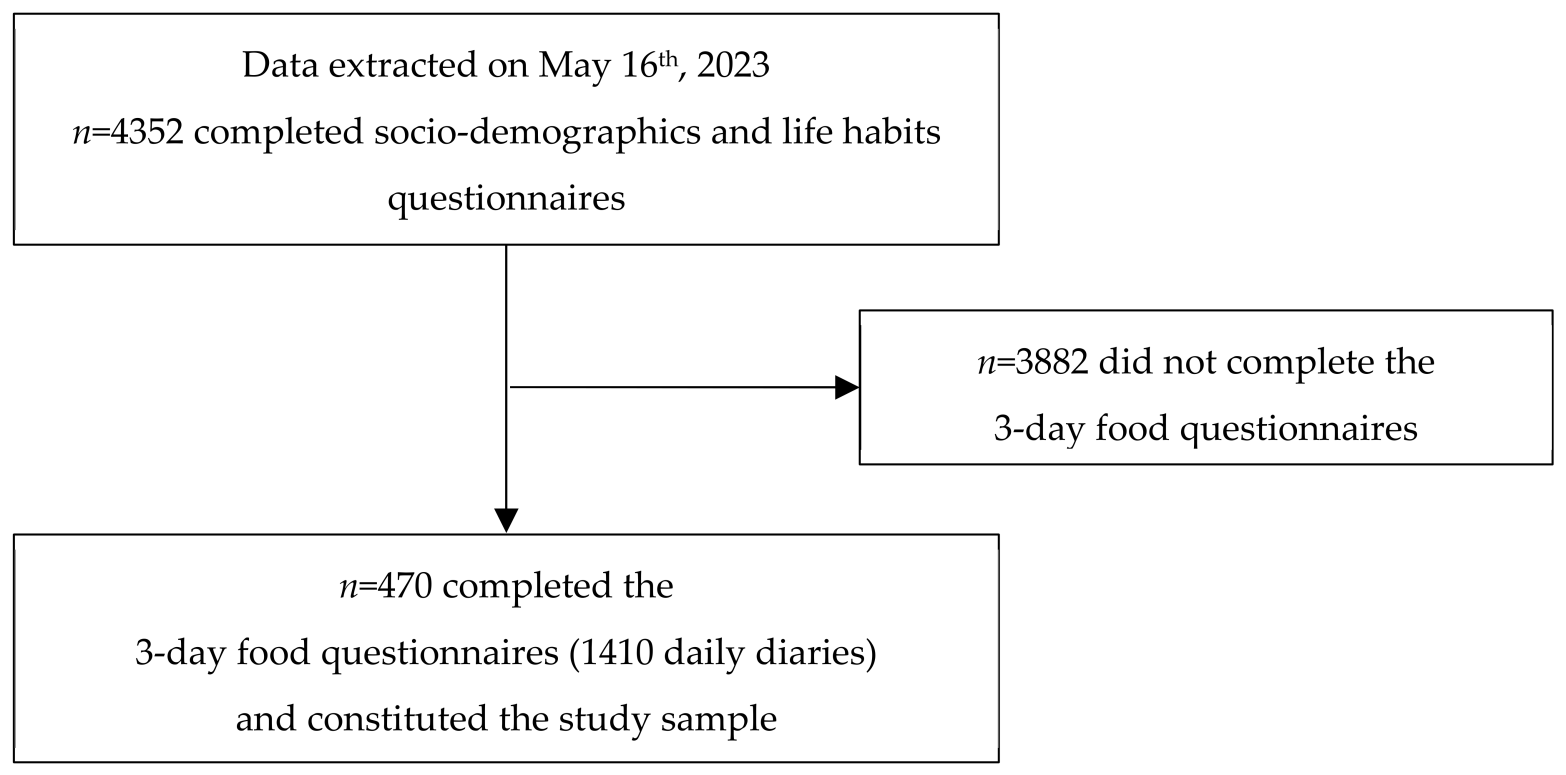
Flowchart of the subjects enrolled in the INVITA study and of the sample selected for this analysis.

Data were extracted from the INVITA survey on 16 May 2023 and included 4352 participants who completed the socio-demographics and life habits questionnaires. Of these, 470 participants (10.8%) completed the 3-day food questionnaires (1410 daily diaries) (Figure 2), all meeting the inclusion criteria set by Willett for normal energy intakes (range of 500–3500 kcal/day for women and of 800–4000 kcal/day for men) [22].

Additionally to the Willett criteria for identification of under-reporters ([22], potential under-reporting of dietary intake was also assessed by calculating the ratio of reported energy intake to basal metabolic rate (EI/BMR). Basal metabolic rate was estimated using the Schofield equation [23]. An EI/BMR ratio of <1.2 allows us to classify participants as potential under-reporters, as this threshold represents the minimum plausible energy intake required to sustain life at the lowest level of physical activity [24]. No participants fell under this threshold. When the more stringent EI/BMR threshold of 1.35, corresponding to the minimum plausible energy intake for a lightly active lifestyle, was additionally applied [24], only two participants (0.04% of the sample) failed to meet the criteria. Therefore, we chose to perform the analysis on 470 participants.

The four dietary groups (116 MEs, 49 FEs, 116 LOVs, and 189 VNs) did not differ across all sociodemographic characteristics and lifestyle habits. There were no significant differences in age, BMI, gender, marital status, education, employment, alcohol consumption, smoking, physical activity, prevalence of menopause (only for females), and age at menopause, with all *p*-values above 0.05. The sample was predominantly female, approximately 93%, with a median age of 34 years and a median BMI of 21.1. Most participants had a university degree, approximately 65%, and were employed, approximately 71%. Regarding lifestyle, 93% did not smoke, and almost all participants had no or low alcohol consumption, with only two subjects at risk. For physical activity, approximately 54% were sedentary and 40% were active. Menopausal women represented 17.7% of the total sample. In summary, the sample was well characterized, and there were no differences between dietary groups in baseline variables (Table 1).

PRAL and NEAP showed negative skewness across dietary groups, particularly among LOV and ME participants. Shapiro–Wilk tests indicated significant deviations from normality for both PRAL and NEAP in all dietary patterns (all *p* < 0.05), supporting the use of non-parametric statistical tests.

In the overall population, median (Me, IQR) PRAL and NEAP values were −12.63 (−26.61, −4.91) mEq/day and 25.86 (12.70, 33.79) mEq/day, respectively.

Dietary acid load significantly differed across dietary patterns, with VN participants showing the lowest PRAL and NEAP values (Table 2). Kruskal–Wallis analyses confirmed significant differences among dietary groups for both PRAL (H = 44.16, *p* = 1.39 × 10^−9^) and NEAP (H = 45.22, *p* = 8.31 × 10^−10^), indicating significant differences in dietary acid load distributions across dietary patterns.

Pairwise Dunn post hoc analyses showed that VN participants significantly differed from all other dietary groups for both PRAL and NEAP, consistently displaying the lowest dietary acid load values and the clearest separation from the other dietary patterns (Table 3). In contrast, FE participants did not significantly differ from ME or LOV participants, despite showing numerically lower median PRAL and NEAP values than ME participants. Because Dunn tests compare rank distributions rather than medians alone, the weaker significance observed for FE comparisons likely reflects the combined effect of the smaller FE sample size, greater distribution overlap, and Holm adjustment for multiple comparisons.

In the overall population, both PRAL and NEAP were positively correlated with BMI, although the association was stronger for NEAP (ρ = 0.22, Holm-adjusted *p* = 0.000003) than for PRAL (ρ = 0.10, Holm-adjusted *p* = 0.028).

Stratified analyses conducted within each dietary pattern suggested a tendency toward higher BMI among participants with higher acid load values, although significant within-pattern associations were limited to NEAP, with positive correlations observed among ME (ϱ = 0.31, Holm-adjusted *p* = 0.005) and LOV participants (ϱ = 0.36, Holm-adjusted *p* = 0.0007). No significant within-pattern correlations were observed for PRAL, nor for either acid load index among FE or VN participants (Table 4). The weaker and less consistent within-pattern correlations may partly reflect the reduced sample size after stratification, together with the narrower range of dietary acid load values within each dietary pattern, which limits statistical power to detect associations. This interpretation is particularly relevant for FE and VN participants, where no significant correlations were observed despite the overall population-level association.

To evaluate the independent association between dietary acid–base load and BMI, we performed multiple linear regression analyses. Because PRAL and NEAP are highly correlated indices of acid–base balance, they were evaluated in separate models. We adopted a progressive adjustment strategy to assess the robustness of the associations against potential confounders.

Initially, we fitted basic models (Model 1) adjusted for total energy intake and dietary pattern. In these basic models, NEAP was significantly associated with BMI (β = 0.034, 95% CI: 0.018 to 0.050; *p* = 0.00005), whereas PRAL was not (β = 0.001, 95% CI: −0.007 to 0.027; *p* = 0.247) (Table 5).

To account for potential lifestyle and demographic confounders, we subsequently fitted fully adjusted models (Model 2), which additionally included age, sex, physical activity level (PAL), smoking status, and education level. In the fully adjusted models, the association between NEAP and BMI remained highly significant and robust (β = 0.032, 95% CI: 0.016 to 0.049; *p* = 0.00013), confirming that a higher dietary acid load is independently associated with a higher BMI even after accounting for lifestyle factors. Conversely, PRAL remained non-significantly associated with BMI after full adjustment (β = 0.011, 95% CI: −0.006 to 0.028; *p* = 0.190) (Table 6).

Notably, in the fully adjusted models, age and physical activity emerged as the strongest independent predictors of BMI (*p* = 0.003 and *p* = 0.00002, respectively, in the PRAL model; *p* = 0.0008 and *p* = 0.00002 in the NEAP model), while the categorical dietary patterns (FE, LOV, and VN) did not show independent significant differences in BMI when compared to ME, once the acid–base load and lifestyle factors were accounted for. This indicates that NEAP values largely accounted for the differences in BMI across dietary patterns. In other words, the lower BMI observed in plant-based groups was largely explained by their lower dietary acid load, rather than by the dietary pattern itself. Specifically, an increase of 10 mEq/day in NEAP corresponded to an average increase of approximately 0.3 kg/m^2^ in BMI. However, because NEAP includes an anthropometric component based on body surface area (calculated from height and weight), and BMI is derived from the same variables, the NEAP–BMI association should be interpreted cautiously, as it may partly reflect mathematical coupling rather than a purely diet-derived effect. For this reason, PRAL should be considered the more diet-specific index, while NEAP should be interpreted as a composite marker combining dietary acid load and anthropometric scaling.

Finally, a post hoc power analysis was conducted using G*Power (F tests, linear multiple regression: fixed model, R^2^ deviation from zero) to evaluate the statistical robustness of the four estimated models. All analyses were based on a total sample size of 470 participants and a significance level of 0.05.

The basic models (Table 5) included two continuous predictors (exposure and energy intake) and one categorical predictor (dietary pattern) with four levels (coded as three dummy variables), for a total of five predictors. The PRAL model yielded an observed R^2^ of 0.010 (Cohen’s f^2^ = 0.010), resulting in an achieved statistical power of 41%. This indicates that the study was underpowered to detect the smaller effect of PRAL at this basic adjustment level. Conversely, the NEAP model yielded a higher R^2^ of 0.045 (Cohen’s f^2^ = 0.047), achieving a statistical power of 96% and confirming the robustness of this association.

For the fully adjusted models (Table 6), the number of predictors increased to 12, by adding the continuous variable age, and the categorical variables sex, physical activity, smoking status, and education level, coded as nine dummy variables. In these models, the achieved statistical power reached 99.8% for the PRAL model (R^2^ = 0.085, Cohen’s f^2^ = 0.093) and 99.9% for the NEAP model (R^2^ = 0.111, Cohen’s f^2^ = 0.125). Notably, while the NEAP model was already robust at the basic adjustment level (96% power), the addition of covariates dramatically increased the power of the PRAL model (from 41% to 99.8%) and its explained variance (from R^2^ = 0.010 to 0.085). This substantial gain in the PRAL model was largely driven by the strong explanatory contribution of the added covariates, particularly age and physical activity.

Although the fully adjusted models showed high overall statistical power (>99.8%), this was largely driven by the strong explanatory contribution of age and physical activity rather than by PRAL itself. The independent effect of PRAL on BMI was very small (β = 0.011), and the study was therefore underpowered to detect this specific effect at the basic adjustment level (41% power for Model 1). For this reason, the non-significant association between PRAL and BMI should be interpreted as exploratory, and future studies with larger sample sizes are needed to clarify whether PRAL has a clinically meaningful independent association with BMI.

Finally, it should be noted that the stratified Spearman correlation analyses conducted within each dietary pattern were subject to reduced statistical power due to the smaller sample sizes, particularly for the FE group (*n* = 49). This limited power, combined with the narrower range of dietary acid load values within each dietary pattern, likely contributed to the lack of significant within-pattern correlations observed for PRAL and for both acid load indices among FE and VN participants, despite the significant population-level associations.

## 4. Discussion

The results of cross-sectional studies [25,26,27,28,29,30,31] and meta-analyses of observational [32,33] and intervention studies [34,35,36,37] are consistent with favorable effects of plant-based dietary patterns on BW.

The acid load of the diet has been shown to affect BW, and the alkalizing effect of a diet may promote weight loss independently from other already well-established mechanisms, like chronic inflammation, oxidative stress, energy imbalance, and the modulation of the microbiota [38].

Dietary acid load has also been associated with other health conditions like insulin resistance, diabetes, hypertension, metabolic syndrome, non-alcoholic hepatic steatosis, kidney diseases, increased bone resorption, reduced bone mineral density, and loss of muscle mass [8,9,10,11,12].

A recent meta-analysis found that PRAL and NEAP were associated with cardiometabolic risk factors, including systolic and diastolic blood pressure and waist circumference. There was a significant difference in the upper and lower categories of PRAL and NEAP, while the lowest versus the highest categories of dietary PRAL were associated with lower BMI and TG levels. The authors concluded that dietary acid load can be considered an independent risk factor for increased anthropometric indices, blood pressure, and TG levels [39].

In our study, the stepwise decrease in animal food consumption across the four dietary patterns (ME → FE → LOV → VN) was associated with a progressive reduction in dietary acid load, as assessed by both PRAL and NEAP, with VN participants showing the lowest values.

At the population level, both PRAL and NEAP were positively correlated with BMI, although the association was stronger for NEAP. In the basic regression models (adjusted for dietary pattern and energy intake), NEAP remained significantly associated with BMI, whereas PRAL did not. To account for potential lifestyle and demographic confounders, we subsequently fitted fully adjusted models including age, sex, physical activity, smoking status, and education level. In these fully adjusted models, the association between NEAP and BMI remained highly significant and robust, confirming that a higher dietary acid load is independently associated with a higher BMI even after accounting for lifestyle factors. Conversely, PRAL remained non-significantly associated with BMI after full adjustment. Notably, age and physical activity emerged as the strongest independent predictors of BMI in both models, while the categorical dietary patterns did not show independent significant differences in BMI once the continuous acid–base load and lifestyle factors were accounted for. This indicates that NEAP values largely accounted for the differences in BMI across dietary patterns; in other words, the lower BMI observed in plant-based groups was largely explained by their lower dietary acid load, rather than by the dietary pattern itself.

Our results, obtained from a sample of Italian adults following ME, FE, LOV and VN dietary patterns, whose eating habits could be influenced by the Mediterranean culture, abundant in fruit, vegetables and grains, are consistent with previous research performed both in Mediterranean and non-Mediterranean cultures. In their cross-sectional study performed in Northern Italy, Sanz et al. found that despite Mediterranean diet adherence being negatively associated with PRAL and NEAP, only dietary acid load, especially assessed using PRAL, but not Mediterranean diet adherence, was associated with indices of metabolic and CV health state [40]. A cross-sectional study performed on 346 Greek students found a higher dietary acid load in subjects with lower adherence to the Mediterranean diet, with a positive association between dietary acid load and central obesity [41]. Our recent investigation performed on a Russian sample established that urinary pH levels were significantly lower in MEs relative to VNs and LOVs, with no significant variation between the two plant-based groups, reflecting an enhanced renal compensation to balance the dietary acid load [42]. Storz et al., who compared the dietary acid load of 8398 nonvegetarians (NVs) and 191 LOVs from the United States National Health and Nutrition Examination Survey (2007–2010), reported higher median dietary acid scores in NVs [43]. A cross-sectional study of 221 Venezuelans following a plant-based diet (*n* = 224) found a stepwise reduction in dietary acid load across flexitarian (*n* = 121), LOV (*n* = 74) and VN (*n* = 29) diets, with VNs showing the greatest alkalizing potential, followed by LOVs and flexitarians [44]. In a cross-sectional study of 371 young Iranian women, the associations of PRAL and NEAP with adiposity and cardiovascular biochemical factors were evaluated. After adjustment for several covariates, a positive association between NEAP and, respectively, overweight, obesity, and waist circumference was found, while PRAL was positively associated only with triglyceride (TG) levels [45]. In another Iranian cross-sectional study performed on 207 students, Mansordehghan et al. correlated PRAL and NEAP with anthropometric indices. After adjustments, NEAP results were positively associated with BMI and other adiposity parameters (hip circumference, fat mass, and body adiposity index) and negatively associated with total body water and the percentage of fat-free mass [46].

In a German 4-week controlled trial, randomly assigning 45 omnivorous subjects to a VN (*n* = 23) or a meat-rich (*n* = 22) diet, dietary acid load significantly decreased in the VN group, who reduced protein and phosphorus intakes [47]. Kahleova et al., in their 16-week randomized clinical trial performed in USA on 244 overweight adults, reported a significant decrease in PRAL and NEAP in the VN group (*n* = 122), while no changes were observed in the control group (*n* = 122). Both scores correlated positively with changes in body weight (BW) and body composition (fat mass and visceral fat), after adjustment for energy intake [48]. In a 12-week randomized clinical trial conducted in US patients with type 1 diabetes, participants assigned to a no-limit low-fat VN diet (*n* = 29) showed a significant reduction in both PRAL and NEAP, when compared to the portion-controlled diet participants (*n* = 29), who had limited energy and controlled carbohydrate intakes. Changes in the dietary acid load were significantly associated with reductions in BW, even after adjustment for changes in energy intake [49]. In another 16-week randomized crossover trial performed by the same US research team, 62 overweight adults were randomized to a Mediterranean or a low-fat VN diet, and changes in PRAL and NEAP were compared with changes in BW. The authors found no changes in PRAL and NEAP when subjects were following the Mediterranean diet, while both markers significantly decreased during the VN diet. Changes in BW at the end of the intervention periods were positively associated with changes in PRAL and NEAP in both diets [50].

Evidence that plant foods may significantly reduce the dietary acid load can be supported by different mechanisms. Plant proteins are rich in glutamate, which disperses hydrogen ions during their metabolism, thus reducing the dietary acid load. Furthermore, fruits and vegetables provide potassium salts of metabolizable anions, which consume hydrogen ions and exert an alkalizing effect. Conversely, protein-rich animal foods, by promoting the accumulation of non-metabolizable anions—primarily sulfate and chloride-, increase dietary acid load [51].

Analogously with other authors [45,46], in our cross-sectional study we found a significant association between NEAP and BMI, which persisted even after full adjustment for lifestyle confounders. However, because NEAP includes an anthropometric component derived from body surface area (calculated from height and weight), and BMI is also derived from height and weight, this association may partly reflect mathematical coupling rather than a purely diet-derived effect. This interpretation is further supported by the fact that PRAL, which is based only on nutrient intake and does not include anthropometric components, did not show a significant independent association with BMI in any adjusted model. Therefore, while NEAP is a statistically robust marker in this cohort, PRAL remains the more appropriate and specific index for isolating the purely diet-derived acid load, despite its weaker association with BMI. Moreover, the cross-sectional nature of the study does not allow conclusions regarding causal relationships, because reverse causation cannot be excluded, hence the observed associations should be interpreted as hypothesis-generating and require confirmation in prospective longitudinal studies.

Nevertheless, the association of dietary acid load with BW has already been evaluated in controlled trials, which can more strongly confirm the association of dietary acid load with BW [47,48,49,50].

A potential mechanism by which an increase in dietary acid load may promote BW gain involves the stimulation of glucocorticoid hormone metabolism by the kidney. Glucocorticoids increase ammonia generation from amino acids—mainly from glutamine—and excretion, leading to increased net acid excretion [52,53]. Specifically, during metabolic acidosis, they promote skeletal muscle catabolism and glutamine release. In the kidney, glucocorticoids also stimulate cortical expression of SN1, the principal transporter involved in glutamine uptake by proximal tubular cells. In addition, they enhance proximal tubular ammonia production and secretion via NHE3 [53]. Their increment can also promote insulin-resistance and appetite alteration [11,53].

The adoption of a three-day weighed food diary constitutes a methodological strength of the present study, as this approach enables the collection of more accurate and detailed dietary intake data than those typically obtained through a standard food frequency questionnaire. Moreover, the comparative assessment of four distinct dietary groups is relevant, especially given the inclusion of a relatively large vegan cohort. The sample size was adequate for statistical analyses and enabled the acid load comparison of three-day food diaries across four dietary groups (MEs, FEs, LOVs, and VNs). To our knowledge, this is the first Italian study to compare the dietary acid load of these four dietary patterns.

As discussed in our previous publication [6], one main limitation of our study is the incompleteness of the Italian food database, developed for epidemiological studies, which lacks many micronutrients, especially in plant foods, leading to underestimation of nutrient intake, particularly in LOV and VN diets. Another limitation is that all data were self-reported, which may introduce potential bias. The INVITA participants, recruited using a web-based approach, were also predominantly female (93%), and the high proportion of women may increase the risk of reporting bias, including social desirability bias, potentially leading to underestimation of energy intake [54]. Furthermore, completing a three-day weighed food record may reflect greater diet awareness, adding further bias. Given the low proportion of smoking and alcohol consumption, this sample represents health-conscious subjects; therefore, our findings may not be generalizable to the wider adult Italian population. Additionally, the post hoc power analysis revealed that while the sample size was sufficient to detect the effects of NEAP (96% power in the basic model, >99.9% in the fully adjusted model), it was underpowered to detect the smaller effect of PRAL (41% power in the basic model). For this reason, the non-significant association between PRAL and BMI should be interpreted as exploratory, and future studies with larger sample sizes are needed to further clarify the specific role of dietary acid load estimated by this index. Furthermore, the non-significant association of PRAL with BMI in our fully adjusted models should be interpreted cautiously. While PRAL is the most diet-specific marker, its independent effect size on BMI is very small. The main dietary finding of our study is therefore the significant difference in PRAL and NEAP values between dietary groups, while the direct association of these indices with BMI is weaker and more difficult to interpret due to the mathematical coupling inherent in the NEAP formula and the limited effect size of PRAL.

Finally, because PRAL and NEAP do not account for protein origin, it is not possible to determine whether results for dietary patterns including mostly or exclusively plant protein could be more favorable.

## 5. Conclusions

In our study, the stepwise decrease in animal food consumption across the four dietary patterns (ME → FE → LOV → VN) was associated with a progressive reduction in both PRAL and NEAP values, with VN participants showing the lowest dietary acid load values. This represents the main dietary finding of our study and confirms that plant-based diets, particularly VN and LOV patterns, have a significantly lower acid load compared to omnivorous diets.

At the population level, both PRAL and NEAP were positively correlated with BMI, although the association was stronger for NEAP. In fully adjusted regression models, NEAP remained significantly associated with BMI, whereas PRAL did not show an independent association. However, because NEAP includes an anthropometric component based on body surface area, and BMI is derived from the same variables, this association should be interpreted cautiously, as it may partly reflect mathematical coupling rather than a purely diet-derived effect. For this reason, PRAL should be considered the more diet-specific marker, while NEAP represents a composite index combining dietary acid load and anthropometric scaling. The direct association between dietary acid load and BMI was therefore weaker and more difficult to interpret than the clear differences observed between dietary groups.

Future studies with larger sample sizes are needed to further clarify the specific role of PRAL, which was underpowered in our cohort, and to investigate whether diet-specific reference ranges could improve the comparison of dietary acid load across different dietary patterns.

## Figures and Tables

**Table 1:** Socio-demographic characteristics and life habits of the overall sample, and the four different dietary groups (*n* = 470): (**a**) Socio-Demographic; (**b**) Life Habits; (**c**) Physical Activity Level (PAL); (**d**) Menopause.

Variable	Overall (470)	ME (116)	FE (49)	LOV (116)	VN (189)	*p*-Value	Test
(**a**) Socio-Demographic							
Age (yrs), Me (IQR) [range: 18–82]	34.0 (28.0–46.0)	32.5 (28.0–43.2)	35.0 (28.0–46.0)	33.5 (28.0–43.0)	36.0 (27.0–48.0)	0.525	Kruskal–Wallis
Gender, n (%)						0.225	Chi-square (low expected freq)
Male	35 (7.4%)	6 (5.2%)	1 (2.0%)	10 (8.6%)	171 (90.5%)		
Female	435 (92.6%)	110 (94.8%)	48 (98.0%)	106 (91.4%)	18 (9.5%)		
BMI Me (IQR) [range 14–48]	21.1 (19.6–23.2)	21.2 (19.6–24.0)	20.8 (19.1–22.0)	20.8 (19.7–23.2)	21.3 (19.6–23.3)	0.409	Kruskal–Wallis
Marital Status, n (%)						0.785	Chi-square
Married	136 (28.9%)	35 (30.2%)	13 (26.5%)	37 (31.9%)	51 (27.0%)		
Not Married	334 (71.1%)	81 (69.8%)	36 (73.5%)	79 (68.1%)	138 (73.0%)		
Education, n (%)						0.516	Chi-square
Professional Qualification/ Diploma	167 (35.5%)	35 (30.2%)	17 (34.7%)	42 (36.2%)	73 (38.6%)		
Degree/ Post-Degree	303 (64.5%)	81 (69.8%)	32 (65.3%)	74 (63.8%)	116 (61.4%)		
Occupation, n (%)						0.943	Chi-square
Employed	334 (71.1%)	82 (70.7%)	36 (73.5%)	84 (72.4%)	132 (69.8%)		
Not Employed	136 (28.9%)	34 (29.3%)	13 (26.5%)	32 (27.6%)	57 (30.2%)		
(**b**) Life Habits							
Monthly Alcohol Consumption, n (%)						0.517	Chi-square (low expected freq)
No consumption	198 (42.1%)	54 (46.6%)	19 (38.8%)	52 (44.8%)	73 (38.6%)		
Low/Moderate	270 (57.4%)	62 (53.4%)	30 (61.2%)	64 (55.2%)	114 (60.3%)		
At risk	2 (0.4%)	0	0	0	2 (1.1%)		
Currently Smoking, n (%)						0.476	Chi-square (low expected freq)
No	435 (92.6%)	111 (95.7%)	44 (89.8%)	106 (91.4%)	174 (92.1%)		
Yes	35 (7.4%)	5 (4.3%)	5 (10.2%)	10 (8.6%)	15 (7.9%)		
(**c**) Physical Activity (PAL)							
Sedentary/Light (1.40–1.69):	253 (53.8%)	74 (63.8%)	22 (44.9%)	62 (53.4%);	95 (50.3%)	0.254	Chi-square (low expected freq)
Active/Moderate (1.70–1.99)	186 (39.6%)	35 (30.2%)	22 (44.9%)	47 (40.5%)	82 (43.4%)		
Vigorous (2.00–2.40)	20 (4.3%)	4 (3.4%)	3 (6.1%)	3 (2.6%)	10 (5.3%)		
Very Low (<1.40)	10 (2.1%)	2 (1.7%)	2 (4.1%)	4 (3.4%);	2 (1.1%)		
Very High (>2.40)	1 (0.2%)	1 (0.9%)	0	00	0		
(**d**) Menopause (females only)							
Menopause, n (%) [435 total females]	77 (17.7%)	14 (12.7%)	10 (20.8%)	16 (15.1%)	37 (21.6%)	0.212	Chi-square
Age at Menopause (yrs), Me (IQR) range	50.0 (48.0–52.0) 27–56	51.0 (50.0–52.0) 27–56	51.0 (48.2–52.8) 37–56	50.5 (49.0–52.2) 33–56	49.0 (47.0–51.0) 30–55	0.104	Kruskal–Wallis

**Table 2:** Dietary acid load by dietary pattern. Values are reported as median (IQR). Overall differences among dietary patterns were assessed using the Kruskal–Wallis test.

Dietary Pattern	PRAL (mEq/day) Median (IQR)	NEAP (mEq/day) Median (IQR)
Meat-Eater	−7.45 (−17.60, 2.22)	30.55 (20.45, 41.66)
Fish-Eater	−9.66 (−23.21, −5.08)	29.20 (15.18, 33.56)
Lacto-Ovo-Vegetarian	−12.13 (−21.89, −4.88)	26.44 (16.86, 34.06)
Vegan	−19.75 (−34.30, −8.57)	17.81 (5.62, 29.35)
Overall *p*-value	1.39 × 10^−9^	8.31 × 10^−10^

**Table 3:** Dunn post hoc pairwise comparisons following Kruskal–Wallis tests (Holm-adjusted *p*-values).

Comparison	PRAL *p*	NEAP *p*
ME vs. FE	0.171	0.107
ME vs. LOV	0.017	0.026
ME vs. VN	5.14 × 10^−10^	3.07 × 10^−10^
FE vs. LOV	0.680	0.924
FE vs. VN	0.013	0.022
LOV vs. VN	0.003	0.001

**Table 4:** Spearman correlations between dietary acid load indices and BMI by dietary pattern (Holm-adjusted *p*-values).

Dietary Pattern	PRAL ϱ	PRAL *p*	NEAP ϱ	NEAP *p*
Meat-Eater	0.21	0.15	0.31	0.005
Fish-Eater	–0.04	1.00	0.10	1.00
Lacto-Ovo-Vegetarian	0.19	0.20	0.36	0.0007
Vegan	0.01	1.00	0.13	0.26

**Table 5:** Basic multiple linear regression models for BMI (Model 1). Dependent variable: BMI (kg/m^2^). Models adjusted for total energy intake and dietary pattern. β coefficients with 95% CI.

Independent Variable	PRAL Model β (95% CI)	*p*	NEAP Model β (95% CI)	*p*
Exposure (mEq/day)	0.001 (−0.007, 0.027)	0.247	0.034 (0.018, 0.050)	0.00005
Energy intake (kcal/day)	0.001 (−0.000, 0.002)	0.060	0.001 (−0.000, 0.002)	0.060
Dietary pattern (ref. Meat-Eater)				
Fish-Eater	−1.110 (−2.393, 0.174)	0.090	−0.966 (−2.228, 0.297)	0.133
Lacto-Ovo-Vegetarian	−0.261 (−1.255, 0.733)	0.606	−0.132 (−1.109, 0.845)	0.791
Vegan	−0.288 (−1.206, 0.630)	0.538	0.041 (−0.861, 0.943)	0.929
Model fit (R^2^)	0.010		0.045	

**Table 6:** Fully adjusted multiple linear regression models for BMI (Model 2). Dependent variable: BMI (kg/m^2^). Models fully adjusted for age, sex, physical activity, smoking, education, energy intake, and dietary pattern. β coefficients with 95% CI.

Independent Variable	PRAL Model β (95% CI)	*p*	NEAP Model β (95% CI)	*p*
Exposure (mEq/day)	0.011 (−0.006, 0.028)	0.190	0.032 (0.016, 0.049)	0.00013
Energy intake (kcal/day)	0.0003 (−0.0005, 0.0012)	0.431	0.0005 (−0.0003, 0.0013)	0.254
Age (years)	0.043 (0.014, 0.072)	0.003	0.049 (0.020, 0.077)	0.0008
Sex (female vs. male)	−0.436 (−1.770, 0.898)	0.521	−0.107 (−1.432, 1.218)	0.874
Physical Activity (ref. < 1.40)				
Very High (>2.40)	16.421 (8.932, 23.911)	0.00002	15.245 (7.840, 22.650)	0.00002
Vigorous (2.00–2.40)	−0.228 (−1.954, 1.499)	0.796	−0.289 (−1.992, 1.413)	0.739
Sedentary/Light (1.40–1.69)	0.206 (−0.513, 0.926)	0.573	0.129 (−0.581, 0.839)	0.722
Smoking (yes vs. no)	−0.086 (−1.392, 1.220)	0.897	−0.118 (−1.406, 1.170)	0.858
Education: Prof./Diploma (ref. Degree)	0.350 (−0.387, 1.087)	0.351	0.276 (−0.451, 1.004)	0.456
Dietary pattern (ref. Meat-Eater)				
Fish-Eater	−0.990 (−2.248, 0.269)	0.123	−0.881 (−2.121, 0.360)	0.164
Lacto-Ovo-Vegetarian	−0.168 (−1.143, 0.807)	0.735	−0.029 (−0.990, 0.931)	0.952
Vegan	−0.250 (−1.155, 0.655)	0.587	0.040 (−0.853, 0.932)	0.930
Model fit (R^2^)	0.085		0.111	

## Data Availability

The data presented in this study are available on request from the corresponding author. The data are not publicly available due to privacy constraints.

## Abbreviations

BMI	Body Mass Index
BW	Body Weight
CV	Cardiovascular
FE	Fish-Eater
IQR	Interquartile Range
LOV	Lacto-Ovo-Vegetarian
ME	Meat-Eater
Me	Median
NEAP	Net Endogenous Acid Production
NV	Non-Vegetarian
PRAL	Potential Renal Acid Load
TG	Triglycerides
VN	Vegan
